# A population-based survey of autistic traits in Kenyan adolescents and young adults

**DOI:** 10.4102/sajpsychiatry.v28i0.1694

**Published:** 2022-02-14

**Authors:** Daniel Mamah, Victoria Mutiso, Isaiah Gitonga, Albert Tele, David M. Ndetei

**Affiliations:** 1 Department of Psychiatry, Washington University, St. Louis, United States of America; 2 Africa Mental Health Foundation, Nairobi, Kenya

**Keywords:** autism, autistic, traits, Africa, Kenya, adolescents, adults

## Abstract

**Background:**

To date, there have been no large-scale population studies of autistic traits (AUT) conducted in Africa.

**Aim:**

The study aimed to estimate the prevalence and characteristics of autism spectrum disorders in a large sample of Kenyan adolescents and young adults.

**Setting:**

Tertiary academic institutions (87%) and directly from the community (13%).

**Methods:**

Our study surveyed 8918 youths (aged 15–25 years) using the autism spectrum quotient (AQ). Based on AQ scores, we derived groups with low (L-AUT), borderline (B-AUT), and high (H-AUT) autistic traits. Relationships of AUT with demographic factors, psychosis, affectivity and stress were investigated.

**Results:**

Internal consistency of the AQ in the population was excellent (Cronbach’s α = 0.91). Across all participants, 0.63% were estimated as having H-AUT, while 14.9% had B-AUT. Amongst community youth, prevalence of H-AUT was 0.98%. Compared to those with low and borderline traits, H-AUT participants were more likely to be males, to have lower personal and parental educational attainment, and to be of a lower socioeconomic status. The H-AUT group also had higher psychotic and affective symptoms as well as higher psychosocial stress than other groups.

**Conclusion:**

The prevalence of H-AUT amongst Kenyan youth is comparable to Autism spectrum disorder (ASD) rates in many countries. Autistic traits in Kenya are associated with worse social and clinical profiles. Further research on autism across Africa is needed to investigate cross-cultural heterogeneity of this disorder, and to guide healthcare policy.

## Introduction

Autism spectrum disorder (ASD) is a neurodevelopmental syndrome characterised by social and communication deficits, as well as restrictive or repetitive behaviours. Symptoms are usually first noticed in early childhood, and occurs three to four times more frequently in boys than in girls.^1^ In developed countries, the prevalence of ASD has doubled over the last two decades to 1.5% of children.^2^ The reasons for this increase are unclear, and may be partly related to improved case identification and diagnostic trends.^2,3^ Methods for determining autism prevalence also varies across studies, which can profoundly affect the estimates. Autism spectrum disorder prevalence has generally been estimated using three basic approaches: tallying diagnosed cases, examining records to also identify undiagnosed cases, and screening large populations. The last method typically produces the most reliable and the highest prevalence estimates.^4^

Autism spectrum disorder has not been extensively studied in Africa which presents a significant gap in our understanding of the global burden of these disorders.^3,5^ Africa has over 1.2 billion people, and about 40% of these are children younger than 14 years old.^6^ In many parts of Africa, those with autism or related developmental disabilities are socially isolated, often using extreme measures, fearing stigma, which casts disabilities as the sign of curse or possession by a spirit.^7,8^ Across Africa, few clinicians have the skills or experiences to identify ASDs. In 2015, there were about 50 child and adolescent psychiatrists for an estimated one billion inhabitants in sub-Saharan Africa.^7^ Thus, only the most severely affected children tend to be diagnosed in Africa, if at all. If diagnosed, this occurs at around age 8, about 4 years later than their counterparts in the United States (US). More than half of African children diagnosed with autism are also found to have an intellectual disability, compared with about one-third of American autistic children, and are more likely to be non-verbal.^7,8^ Considering that national health policies for children with ASD is largely absent in most African countries, estimating the prevalence and characteristics of these disorders in the continent is necessary to appropriately plan intervention strategies for affected individuals and their families.^5^

There is limited data on ASD prevalence in African countries. A study of children born to Somali parents living in Sweden found that rates of ASD was about three times higher than in children of non-Somali parents.^9^

Existing research on ASD has previously been reviewed in Africa, most of which were conducted in South Africa and Nigeria.^10^ Four studies attempted to estimate the burden of ASD in the continent, largely from clinical populations. One study screened 2320 patients at a Nigerian paediatric neurology clinic and found a 2.3% prevalence of ASD.^11^

Another group studied 44 Nigerian children with intellectual disorders, 11.4% of whom had an ASD.^12^ In an older survey, involving children from six African countries, Lotter reported an ASD prevalence of 2.3%, and also noted lower occurrence of ritualistic/repetitive behaviour in these patients compared to British patients.^13^ In this study by Lotter, however, facilities identified as having mentally disabled children were evaluated, and thus did not represent a general population survey. The only community survey of ASD in sub-Saharan Africa to our knowledge, involved 1169 children in Kampala, Uganda.^14^ These authors found an ASD prevalence of 0.68% in Kampala, consistent with the median ASD prevalence (0.62%) from a systematic review of international epidemic surveys.^3^ Notably, this is lower than the 1.7% ASD prevalence reported in the US.^15^

Estimating ASD rates in children can limit its use in developing countries as it often requires extensive interviews. Self-report questionnaires can be useful in older populations to estimate the prevalence of autistic traits (AUT), even though they are not a substitute for clinical evaluation of ASD. Our current study explores the prevalence of AUT using the *autism spectrum quotient* (AQ)^16,17^ in a large (*N* = 8918) cohort of Kenyan adolescents and young adults. The majority (87%) of those surveyed were tertiary school students, facilitating the recruitment of a very large population sample who can reliably complete the questionnaires. We also explore the relationship of AUT to clinical and sociodemographic characteristics.

## Research study design and methods

### Population and setting

Participants were recruited from Nairobi county which is largely urban and Machakos, Kitui and Makueni counties, largely rural areas in Kenya. The majority of participants (87%) were recruited from tertiary academic institutions (i.e. eight colleges and one public university), whilst 13% were recruited directly through community outreach. University and college students across a range of disciplines were approached in their classrooms, with permission of school authorities. Community youth were directed to specific public meeting areas for assessments, with the help of local community leaders. Participation in the study was voluntary, and participants were not given monetary compensation because of the local regulations. In some cases, snacks and drinks were provided. Inclusion criteria consisted of being aged 15–25 years, and having the ability to speak, read and write English.

### Ethical considerations

The study was approved by the ethical review board of Maseno University and the Institutional Review Board of Washington University in St. Louis. A written and verbal informed consent to participate in the study was obtained from all participants and from parents/guardians of those below the age of 18.

### Clinical and demographic assessments

Participants completed the adolescent AQ^16,17^ to evaluate social functioning and AUT. The AQ is a self-assessment questionnaire that covers five different autistic domains: (1) social skills, (2) communication skills, (3) imagination, (4) attention to detail, and (5) attention switching/tolerance to change.^18^ Autism spectrum quotient scores range between 0 and 50, with a score of 32 or higher indicative of a strong likelihood of an ASD, and scores of 26–31 suggesting a borderline indication of ASD.^17,18^

Participants completed a demographic questionnaire, which included questions on household items, water source, floor type, toilet type and cooking method, which have been used to estimate economic status^19^ (see Table 1). Participants also completed the Washington Early Recognition Center Affectivity and Psychosis (WERCAP) screen^20,21^ which quantitatively assesses psychosis-risk symptoms and bipolar-risk symptoms (‘affectivity’) based on symptom frequency and effects on functioning,^20^ and has shown high test-retest reliability and validity.^20^

**TABLE 1:** Demographic characteristics of participant groups (*N* = 8918).

Characteristic	H-AUT		B-AUT		L-AUT		H-AUT vs. L-AUT)		
	*N*	%	*N*	%	*N*	%	d/V†	F/chi-sq.	*p*
**Total**	56	0.63	1332	14.9	7530	84.4	N/A	-	-
**Age (s.d.)**	20.97	2.4	20.95	2.3	21.25	1.9	0.13	12.8	< 0.0001*
**Gender**							**0.20**	**18.9**	< 0.0001 *
Female	17	30.4	559	42.1	3547	47.5	-	-	-
Male	39	69.6	768	57.9	3924	52.5	-	-	-
**Education** ‡							**0.58**	**109.0**	< 0.0001*
Primary school	7	12.5	167	12.6	476	6.3	-	-	-
Secondary school	2	3.6	89	6.7	353	4.7	-	-	-
College, Tech. or Prof. Sch.	14	25.0	256	19.2	1287	17.1	-	-	-
Undergraduate university	16	28.6	364	27.4	2591	33.1	-	-	-
Graduate university	17	30.4	445	33.4	2888	38.4	-	-	-
**Employment status**							**0.34**	**64.8**	< 0.0001*
Employed							-	-	-
Self-employed	3	5.4	92	6.9	383	5.1	-	-	-
Part-time	1	1.8	11	0.8	43	0.6	-	-	-
Full-time	1	1.8	26	2.0	39	0.5	-	-	-
Unemployed	8	14.3	213	16.0	919	12.2	-	-	-
Student	43	76.8	972	73.0	6071	80.7	-	-	-
**Marital status**							0.06	9.7	0.14
Married	2	3.6	94	7.1	389	5.2	-	-	-
Single	54	96.4	1225	92.5	7097	94.5	-	-	-
Widowed	0	-	2	0.2	7	0.1	-	-	-
Divorced	0	-	4	0.3	14	0.2	-	-	-
**Religion**							0.06	10.3	0.11
Protestant Christian	35	62.5	719	54.2	4298	57.6	-	-	-
Catholic Christian	17	30.4	491	37.0	2557	34.3	-	-	-
Muslim	4	7.1	56	4.2	326	4.4	-	-	-
Other	0		60	4.5	280	3.8	-	-	-
**Position of birth**	**2.55**	**1.6**	**2.71**	**1.9**	**2.76**	**1.9**	0.10	0.6	0.57
**Sibling size**	**4.32**	**3.4**	**4.19**	**3.1**	**4.18**	**3.2**	0.06	0.07	0.93
**Maternal education** ‡							**0.19**	**40.2**	0.0002*
Primary school	15	26.8	250	18.8	1367	18.2	-	-	-
Secondary school	9	16.1	259	19.5	1453	19.3	-	-	-
College, Tech. or Prof. Sch.	14	25.0	247	18.6	1492	19.8	-	-	-
Undergraduate university	8	14.3	292	21.9	1726	22.9	-	-	-
Graduate university	3	5.4	123	9.2	872	11.6	-	-	-
Unknown	7	12.5	160	12.0	616	8.2	-	-	-
**Paternal education** †							**0.25**	**52.1**	< 0.0001*
Primary school	14	25.0	178	13.4	950	12.6	-	-	-
Secondary school	7	12.5	205	15.4	1093	14.5	-	-	-
College, Tech. or Prof. Sch.	17	30.4	275	20.7	1703	22.7	-	-	-
Undergraduate university	8	14.3	303	22.8	1693	22.5	-	-	-
Graduate university	4	7.1	153	11.5	1201	16.0	-	-	-
Unknown	6	10.7	216	16.2	876	11.7	-	-	-
**Parents marital status (%)**							**0.10**	**15.7**	0.02*
Married	45	80.4	1071	80.6	5956	79.5	-	-	-
Widowed	2	3.6	117	8.8	853	11.4	-	-	-
Divorced	5	8.9	74	5.6	342	4.6	-	-	-
Never married	4	7.1	67	5.0	341	4.6	-	-	-
**Home residents**									
Mother	22	39.3	772	58.0	4452	59.1	**0.10**	**9.5**	0.009*
Father	3	5.4	134	10.1	716	9.5	0.02	1.6	0.46
Sibling(s)	1	1.8	39	2.9	225	3.0	0	0.3	0.87
Spouse/partner	9	16.1	329	24.7	2206	29.3	**0.17**	**16.0**	**0.0003**
Child(ren)	5	8.9	146	11.0	748	9.9	0.01	1.4	0.50
Grandparent(s)	8	14.3	153	11.5	890	11.8	0.01	0.5	0.79
Other family member(s)	4	7.1	52	3.9	358	4.8	0.03	2.6	0.27
Friend	6	10.7	65	4.9	403	5.4	0.04	3.7	0.15
Other	1	1.8	2	0.2	50	0.7	0.07	6.4	0.04*

H-AUT, high autistic traits; B-AUT, borderline autistic traits; L-AUT, low autistic traits; s.d., standard deviation.

Values are given as means (s.d.) or number per group (%). Results derived from results of two-sided ANOVA tests or Chi-Square analyses.

† , *d* = Cohen’s d, comparing H-AUT and L-AUT groups. V = Cramer’s V, involving all three groups. Cohen’s d was used for determining effect size of continuous variables, and Cramer’s V for categorical variables. Values of Cohen’s d and Cramer’s V cannot be directly compared.

‡ , Education indicated as years of schooling.

* , *p* < 0.05.

The WERC Stress Screen, a self-report questionnaire, was used to assess total stress burden and the severity of individual stressors.^20,21^

### Statistical analysis

All statistical analyses were carried out using SAS 9.4 (SAS Institute Inc., Cary, NC). Participants were grouped based on scores on the AQ, in line with previously described provisional diagnostic criteria^17,18^: high AUT (H-AUT; > 32), borderline AUT (B-AUT; 26–31), and low AUT (L-AUT; < 25).

Effect sizes were measured for clinical and demographic variables across groups. *Cohen’s d* was used to calculate effect size for continuous variables, and *Cramer’s V* for categorical variables. Chi-square and two-sided analysis of variance (ANOVA) tests to estimate statistical significances. Groups were also compared based on scores on the five derived areas on the AQ: social skill, attention switching, attention to detail, communication and imagination.^18^ Internal consistency of the AQ was assessed using Cronbach’s alpha (α).

## Results

### Sample characteristics

Participants approached in the classrooms or designated public areas all consented to participating in the study, and there were no youth reporting a refusal to participate. A total of 8918 adolescents and young adults met age (15–25 years) and other inclusion criteria, and participated in our study. The average age (standard deviation [s.d.]) of the respondents was 21.2 (2.0) years, with a median of 21.3 years. There were 4123 females (46.6%) and 4731 males (53.4%) in the study sample.

The average age (s.d.) of tertiary school students was 21.4 (1.7) years compared to 19.6 (2.9) years for community participants (*t* = 30.6; *p* < 0.0001). The percentage of females and males were 47.9% and 52.1% in students and 37.6% and 62.4% in community participants, respectively (χ^2^ = 41.7; *p* < 0.0001).

### Internal consistency of the autism spectrum quotient

The internal consistency of the total AQ score was excellent across the entire study population (Cronbach’s α = 0.91).

### Gender effects on autistic traits

**FIGURE 1:**
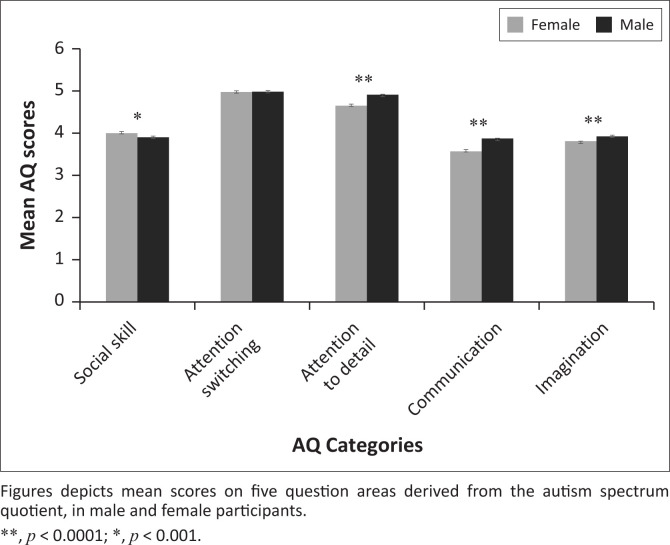
Sex differences of autistic traits in Kenya.

The average (s.d.) score on the AQ was 21.28 (4.3) across all participants. Average AQ scores in females was 21.00 (4.2) and 21.52 (4.3) in males. Group differences were statistically significant (*d* = 0.12; *t* = 5.8, *p* < 0.0001). Figure 1 shows average scores across the AQ categories by gender. Results showed males had greater impairment than females on *communication* (*d* = 0.153; *t* = 7.2, *p* < 0.0001), *attention to detail* (*d* = 0.134; *t* = 6.3, *p* < 0.0001), and *imagination* (*d* = 0.084; *t* = 4.0, *p* < 0.0001). Females had greater impairment than males on *social skills* (*d* = 0.07; *t* = −3.4, *p* = 0.0007). There were no significant group effects for *attention switching* (*d* = 0.0; *t* = 0.0, *p* = 0.98).

### Age effects on autistic traits

There was a small age effect of total AQ scores, with slightly decreased scores in older individuals (*r* = −0.08; *p* < 0.0001). Similarly, slightly decreased AQ scores with age were found for *social skill* (*r* = −0.09; *p* < 0.0001), and *communication* (*r* = −0.08; *p* < 0.0001), while decreased AQ scores with age for *attention switching* (*r* = −0.02; *p* = 0.06) trended towards significance. There was no significant age relationship with either *attention to detail* (*p* = 0.32) or *imagination* (*p* = 0.14).

### Demographic characteristics of autism-risk groups

Demographic and economic data for the three autism-risk categories are presented in Tables 1 and 2. Amongst all youths surveyed, 0.63% met criteria for H-AUT based on the AQ (i.e. score ≥ 32), while 14.9% were B-AUT. The proportion of male to female H-AUT participants (70:30) was higher than either the B-AUT (58:42) or L-AUT (53:47) groups.

**TABLE 2:** Economic characteristics of participant groups (*N* = 8918).

Characteristic	H-AUT		B-AUT		L-AUT		(H-AUT vs. L-AUT)		
	*N*	%	*N*	%	*N*	%	d/V†	F/chi-sq	*p*
**Items in household**									
Electricity	31	55.4	752	56.5	4861	64.6	**0.35**	**33.5**	< 0.0001 *
Radio	43	76.8	1058	79.4	6247	83.0	**0.12**	**11.0**	0.004*
Television	30	53.6	744	55.9	4667	62.0	**0.20**	**19.1**	< 0.0001*
Refrigerator	10	17.9	257	19.3	1790	23.8	**0.14**	**13.6**	0.001*
Cell phone	40	71.4	948	71.2	5770	76.6	**0.20**	**18.9**	< 0.0001*
Bicycle	25	44.6	469	35.2	3014	40.0	**0.12**	**11.7**	0.003*
Motorcycle	8	14.3	243	18.2	1560	20.7	0.06	5.5	0.06
Motor vehicle	10	17.9	219	16.4	1502	20.0	**0.10**	**9.0**	0.01*
**Home water source**							**0.11**	**20.5**	0.009*
Piped water	13	23.6	369	27.8	2417	32.3	-	-	-
Public water	7	12.7	190	14.2	1011	13.5	-	-	-
Well water	15	27.3	357	26.9	2076	27.8	-	-	-
Surface water	19	34.6	380	28.6	1847	24.7	-	-	-
Other	1	1.8	31	2.3	127	1.7	-	-	-
**Home floor**									
Earth	14	25.0	365	27.4	1653	22.0	**0.20**	**9.3**	< 0.0001*
Cement	30	53.6	727	54.6	4319	57.4	0.04	3.8	0.15
Tile	11	19.6	190	14.3	1456	19.3	**0.20**	**19.3**	< 0.0001*
Wood	2	3.6	45	3.4	118	1.6	**0.23**	**21.4**	< 0.0001*
Other	0		10	0.8	19	0.3	0.09	8.9	0.01*
**Home toilet**							**0.24**	**38.6**	< 0.0001*
No toilet	0	-	33	2.5	105	1.4	-	-	-
Pit latrine	40	71.4	983	73.9	5589	74.3	-	-	-
Flush toilet	12	21.4	259	19.5	1668	22.2	-	-	-
Other	4	7.1	56	4.2	159	2.1	-	-	-
**Home cooking method**							**0.14**	**30.0**	0.0009*
Firewood	32	57.1	774	58.2	3885	51.6	-	-	-
Charcoal	10	17.9	151	11.3	1077	14.3	-	-	-
Kerosene stone	1	1.8	47	3.5	263	3.5	-	-	-
Gas stove	12	21.4	308	23.1	2020	26.9	-	-	-
Electric stove	1	1.8	29	2.2	202	2.7	-	-	-
Other	0		22	1.7	77	1.0	-	-	-

H-AUT, high autistic traits; B-AUT, borderline autistic traits; L-AUT, low autistic traits.

Values are given as means (s.d.) or number per group (%). Results derived from results of two-sided ANOVA tests or Chi-Square analyses.

† , Cramer’s V used to determine effect size, and reported results are from comparing H-AUT with L-AUT groups.

* , *p* < 0.05.

Large effect sizes were observed for: educational attainment, employment status, and paternal educational attainment. Medium effect sizes were observed for: maternal educational attainment, electricity in the home, type of home toilet, and type of home cooking method. Parental educational attainment was lower in the H-AUT group compared to L-AUT, with B-AUT groups being intermediate. More L-AUT participants were active students compared to those with autistic traits, and generally had higher educational attainment. Compared to L-AUT, fewer of those in the H-AUT group lived in homes with electricity, flush or pit latrines, and electric or gas stoves. Those with H-AUT were also less likely to have piped water in their homes compared to L-AUT subjects, and were less likely to possess motor vehicles, televisions, radios, refrigerators and cell phones.

### Comparison of autism-risk groups in tertiary school students and community youth

**FIGURE 2:**
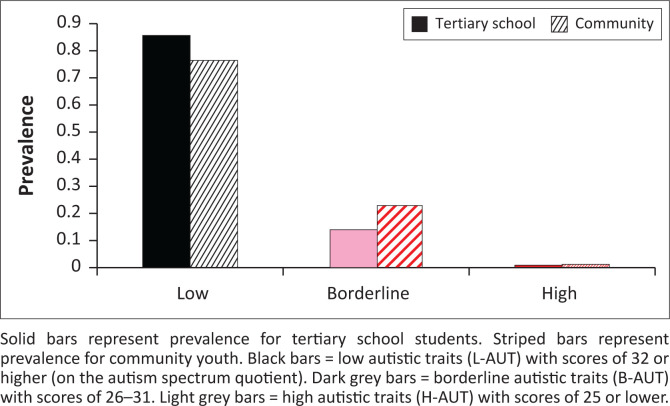
Prevalence of autistic trait categories in tertiary school students and community youth.

Figure 2 shows the percentages of youth who are H-AUT, B-AUT and L-AUT amongst tertiary school students or community participants. Amongst the students, 0.58% met criteria for H-AUT, compared to community youth where prevalence of H-AUT was 0.98%. A chi-square analysis showed significant differences in autism-risk groupings across students and community youth (*χ*^2^ = 62.8; *p* < 0.0001).

### Relationship with psychosis and affectivity

We evaluated the relationship of symptom severity from the WERCAP Screen with AQ symptom scores, and found a significant correlation with both affectivity (*r* = 0.13; *p* < 0.0001) and psychosis (*r* = 0.19; *p* < 0.0001). Based on psychosis scores, six H-AUT participants (10.7%) had severe psychotic symptoms,^20^ compared to 106 (8.0%) of B-AUT, and 307 (4.1%) of L-AUT subjects (χ^2^: 42.6; *p* < 0.0001).

Table 3 shows average affectivity and psychosis scores. Group differences were significant for both affectivity (*F* = 32.9; *p* < 0.0001) and psychosis (*F* = 71.8; *p* < 0.0001). Post-hoc analysis showed affectivity severity was significantly higher in H-AUT than in L-AUT (*d* = 0.41; *t* = −3.1; *p* = 0.002) groups, but there were no significant differences between H-AUT and B-AUT groups (*d* = 0.18; *p* = 0.2). Post-hoc analysis showed psychosis severity was significantly higher in H-AUT participants than in both L-AUT (*d* = 0.56; *t* = −5.1; *p* < 0.0001) and B-AUT (*d* = 0.27; *t* = −2.1; *p* = 0.03) groups.

**TABLE 3:** Clinical characteristics of participant groups (*N* = 8918).

Characteristic	H-AUT		b-AUT		L-AUT		(H-AUT vs. L-AUT)		
	*N*	%	*N*	%	*N*	%	d/V†	F/chi-sq	*p*
**WERCAP**									
Affectivity (chronic)	13.6	8.4	12.0	8.9	10.1	8.3	**0.41**	**32.9**	< 00001 *
Psychosis (chronic)	14.7	13.3	11.4	11.1	8.2	9.5	**0.56**	**71.8**	**< 0**.0001*
Stress	36.7	24.7	32.1	30.3	24.9	26.5	**0.42**	**43.9**	< 0.0001*

H-AUT, high autistic traits; B-AUT, borderline autistic traits; L-AUT, low autistic traits; WERCAP, Washington Early Recognition Center Affectivity and Psychosis.

Values are given as means (s.d.) or number per group (%).

* , *p* < 0.05.

### Relationships to stress

**FIGURE 3:**
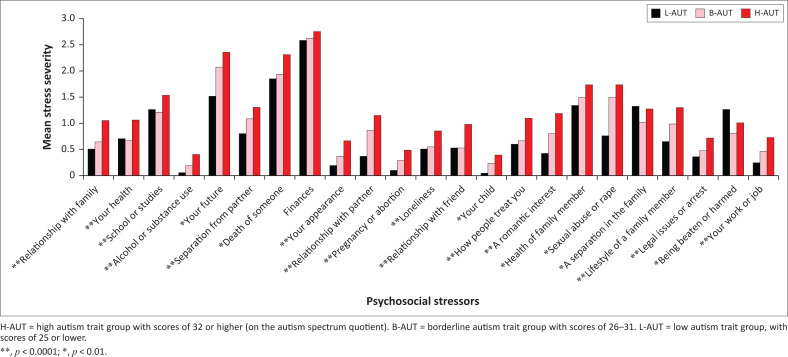
Psychosocial stressors across groups. Figure represent mean item scores on the Washington Early Recognition Center (WERC) Stress Screen in each group.

We found a significant relationship of AQ symptom scores with psychosocial stress severity (*r* = 0.13; *p* < 0.0001). As seen in Table 2, group differences in stress severity were also significant (*F* = 43.9; *p* < 0.0001). Post-hoc analysis showed stress severity was significantly higher in the H-AUT group than in the L-AUT group (*d* = 0.42; *t* = −3.1; *p* = 0.002), but not compared to the B-AUT group (*d* = 0.13; *p* = 0.4). A comparison of severity of individual stressors across groups are shown in Figure 3. Most stressors showed significant group differences, generally with the H-AUT group having the most severe mean score, and B-AUT having intermediate scores between that of H-AUT and L-AUT groups. The largest effect sizes were for stressors involving school/studies, relationship with friends, a romantic interest, and a separation in the family. Highest mean scores for all groups involved financial stress, and there were no significant group differences on this item.

## Discussion

Our study is the first assessing the prevalence of AUT in Kenya. To our knowledge, there are also no other studies investigating these traits in adolescents or young adults in Africa. While our studies did not directly assess autism rates, we found H-AUT in 0.63% of the Kenyan youths surveyed. This prevalence rate is comparable to median rates of ASDs (determined using clinical criteria) amongst countries in a global survey (0.62%), which notably did not include any countries from Africa.^3^ This global survey showed a wide variability in ASD rates across countries. For example, the median ASD prevalence across European countries was about 0.62%, but ranged from 0.3% to 1.16%. Global variability in reported ASD rates is likely influenced by differences in diagnostic criteria and assessment methods used, and awareness of the disorder in both the lay and professional public.^2,3^ Our study highlights that variability in prevalence rates is also affected by the characteristics of the populations studied. We found that youth recruited from the community had higher H-AUT rates (0.98%) than those who are students (0.58%), suggesting that H-AUT are an impediment to higher educational attainment in the population.

We found that H-AUT were 2.3 times more prevalent in male than female subjects. This gender disparity is lower than the 4:1 ratio found in many autism studies, which may reflect differences in the clinical entities captured, as not all high AQ subjects will have an autism diagnosis. However, it has been suggested that there might be a gender bias in autism studies, with girls being less likely to receive a clinical diagnosis than boys, and that a more accurate gender ratio is closer to 3:1.^1^ In Kenya, we found that male subjects endorsed greater impairment on most AQ sub-items, but female subjects had more social skills impairment than boys. The effect sizes of these group differences however were minimal (Cohen’s *d* < 0.2), and thus may not be clinically relevant. Population studies have reported fewer social behaviour problems in girls compared to boys,^22^ which differs from what was observed in our study. It is possible that cultural norms in the communities surveyed in Kenya led to female subjects rating social behaviour items as more abnormal than male subjects. For example, questions probing a ‘preference for isolated activities’, ‘finding it hard to socialise’, or ‘preference of a library over a party’, may be endorsed more by young women in Kenya than in some other cultures, because of conservative attitudes, particularly amongst adolescents.^23^ Additionally, high early motherhood rates in Kenya, with half of young women having given birth by age 20,^24^ can also influence social preferences.

Youth with H-AUT in our study had a lower personal and parental educational attainment than those in other groups, suggesting that AUT are associated with intellectual impairment in many participants. This is supported by previous studies showing intellectual disability or borderline intellectual functioning present in 56% of ASD individuals.^15^ Lower educational attainment in H-AUT youths may be mediated by other characteristics often found to be prevalent in ASD, such as Attention deficit hyperactivity disorder (ADHD) or other behavioural disorders.^25^ Based on possessions and utilities, we were able to estimate the socioeconomic status of our participants. The H-AUT group tended to be poorer than those in the other groups. This is consistent with previous studies showing lower socio-economic status associated with increased autism rates.^26^ Some of the increased poverty with increased autistic trait severity may be secondary to lower educational attainment, which was also seen in our study. Additionally, severe social deficits in those with ASD can limit the types of job opportunities available, and the social connections for advancing in the workplace. Interestingly, in developed nations the prevalence of ASD is often found to correlate with increase socio-economic status because of under-identification of autism in lower income children.^27^

We found a correlation of increased psychotic and affective symptoms with AUT. Psychosis scores were about 80% higher in H-AUT individuals compared to those in the L-AUT group, while affective symptoms were about 35% higher. Increased psychiatric comorbidities are often observed in those with ASD.^28^ For example, the prevalence of schizophrenia in individuals with ASDs is estimated to be about 3.6%,^29^ substantially higher than in the general population, and higher rates of non-affective psychotic disorders in this autism have also been reported.^30^ A diagnostic assessment would be necessary to clarify the rates of psychiatric disorders in our study population, since high scorers on the psychosis screen do not necessarily have an existing psychotic disorder. However, more extensive psychotic experiences increase the likelihood of having early schizophrenia or bipolar disorder.^21^ We also found that H-AUT participants had increased affective symptoms and greater stress severity compared to the L-AUT group. This is consistent with previous observations of high levels of depression, anxiety and stress in adult ASD individuals.^31,32^ High levels of comorbid psychiatric symptoms and stress are associated with greater disability,^31^ and underscore the need for identification and specialised treatment of those with autistic symptoms in Kenya. Autism spectrum disorder is often underdiagnosed in adults, or overlooked when psychiatric comorbidities are present.^33^

Investigating AUT in Kenya provides insights into how widespread autism may be in the African population, and underscores the need for health policies that include neurodevelopmental disorders. Many children with ASD in Africa are socially isolated, often using extreme measures, and are never diagnosed or receive treatment.^7^ In many parts of the continent, developmental disabilities carry a societal stigma and often are attributed to a curse (e.g. brought on by a taboo act, such as cheating on a spouse) or evil possession.^7,8^ Much of this is linked to the significant unawareness of autism in the community in Africa, and affected individuals are often not brought for treatment. In Kenya, there are no diagnostic facilities for autism related disorders, and no intervention guidelines exist.^34^ Screening for AUT, as we have performed in this study, could be used in early identification of cases who may require further evaluation.

Some limitations should be considered with our study. Study participation was limited to those who are fluent in English, thus it is possible that results of our study, particularly those surveyed directly from the community, are not representative of the population. However, the vast majority of Kenyans within the study age range, and almost all those attending tertiary institutions are fluent in English, and thus, unlikely to substantially affect the results. Our findings are also not representative of the entire Kenyan youth population, but rather represents a subset of the population most of whom attend tertiary institutions. Many intellectually disabled individuals and those with low-functioning individuals would not be captured in our survey, and thus our survey likely underestimates the prevalence of AUT. This is supported by our finding of more H-AUT youths amongst the community sample compared to the student sample. While high scorers on the AQ have been found to correlate with ASD diagnosis, the AQ is an imprecise estimate of ASD.^35^ Clinical assessments, often involving information from collateral sources, are considered the standard of care for estimating ASD prevalence rates. Therefore, our results do not directly indicate rates of autism in the community. The validity of the AQ in African populations is also unclear, as this is the first study using this questionnaire in the continent. However, the AQ has been validated across multiple different cultures, including in Japan,^36,37^ the Netherlands,^38^ Australia,^39^ French-Canada,^40^ and the United Kingdom,^18^ and thus appears to have some cross-cultural validity. Furthermore, excellent internal consistency of the AQ suggests that it is measuring a general autistic construct in the Kenyan population. Nevertheless, there may be culture-specific traits in Kenya that may be erroneously captured as abnormal with the AQ. Future studies validating the AQ against clinician assessment in Kenya would be important in interpreting the AQ in this population. Also, estimating symptom prevalence using culturally appropriate screening tools or clinical examination may help validate our findings.^14^

## Conclusion

In summary, we present the first epidemiologic study of AUT in Kenya. We found the prevalence of H-AUT to be 0.63% of adolescents and young adults. Autistic traits were related to lower educational attainment, lower socioeconomic status as well as psychosis, mood symptoms, and stress. The lack of autism research in Africa suggests a critical need for further capacity building. Increased awareness and education about autism in Kenya are expected to lead to improved help-seeking behaviour and mental health policies.
